# Epidermal growth factor receptor (*EGFR*) mutations and expression in squamous cell carcinoma of the esophagus in central Asia

**DOI:** 10.1186/1471-2407-12-602

**Published:** 2012-12-17

**Authors:** Behnoush Abedi-Ardekani, Nazir Ahmad Dar, Mohammad Muzaffar Mir, Showkat Ahmad Zargar, M Muqbool Lone, Ghyslaine Martel-Planche, Stéphanie Villar, Mounia Mounawar, Farrokh Saidi, Reza Malekzadeh, Pierre Hainaut

**Affiliations:** 1 International Agency for Research on Cancer, Lyon, France; 2 Digestive Disease Research Center, Shariati Hospital, Tehran University of Medical Sciences, Tehran, Iran; 3 Social security Organization, Tehran, Iran; 4 Department of Biochemistry, University of Kashmir, Srinagar, India; 5 Departments of Clinical Biochemistry, SK-Institute of Medical Sciences, Soura Srinagar, JK, India; 6 Department of Gastroenteriology, SK-Institute of Medical Sciences, Soura Srinagar, JK, India; 7 Department of Radiation Oncology, SK-Institute of Medical Sciences, Soura Srinagar, JK, India; 8 College of Medicine, Al Jouf University, Sakaka, Al Jouf, 75471, KSA; 9 Institute for Cancer Research, Chester Beaty Laboratories, London, UK; 10 International Prevention Research Institute, Lyon, France

**Keywords:** Squamous cell carcinoma, Esophagus, *EGFR* mutations, Golestan, Kashmir

## Abstract

**Background:**

Esophageal squamous cell carcinoma (ESCC) shows geographic variations in incidence, with high incidences (>50/10^5^ person-years) in central Asia, including North Eastern Iran (Golestan) and Northern India (Kashmir). In contrast to Western countries, smoking does not appear to be a significant risk factor for ESCC in central Asia. In lung adenocarcinoma, activating mutations in the gene encoding epidermal growth factor receptor (*EGFR*) are frequent in tumors of never smokers of Asian origin, predicting therapeutic sensitivity to *Egfr*-targeting drugs.

**Methods:**

In this study 152 cases of histologically confirmed ESCC from Iran (Tehran and Golestan Province) and North India (Kashmir Valley) have been analyzed for *EGFR* mutation by direct sequencing of exons 18–21. *Egfr* protein expression was evaluated by immunohistochemistry in 34 samples from Tehran and *HER2* mutations were analyzed in 54 cases from Kashmir.

**Results:**

A total of 14 (9.2%) *EGFR* variations were detected, including seven variations in exons. Among those, four (2.6%) were already documented in lung cancers, two were reported as polymorphisms and one was a potentially new activating mutation. All but one variation in introns were previously identified as polymorphisms. Over-expression of *Egfr* was detected in 22/34 (65%) of tested cases whereas no *HER2* mutation was found in 54 cases from Kashmir.

**Conclusion:**

Overall, *EGFR* mutations appear to be a rare event in ESCC in high incidence areas of central Asia, although a very small proportion of cases may harbor mutations predicting sensitivity to anti-*Egfr* drugs.

## Background

Esophageal cancer is the eighth most common cancer and sixth cause of cancer death worldwide, with the majority of cases occurring in low and middle resource countries [1]. Esophageal Squamous Cell Carcinoma (ESCC) represents about 80% of the cases worldwide and is by far the most common histological type in low-resource countries, whereas adenocarcinoma represents 20-50% of the cases in some Western countries [2]. There are striking geographic variations in incidence. Very high incidence rates have been consistently reported in a region of central Asia that extents from the Caspian Sea to central China, defining the so called “*Asian Esophageal Cancer Belt*” [3,4]. North eastern Iran (Golestan) and Northern India (Kashmir Valley) are part of this high incidence region. Current cancer registration in Golestan shows incidence rates of about 50/10^5^ person-years in both genders [5,6]. Although there is no continuous cancer registration in Kashmir, observational studies suggest incidence rates of 42 and 27/10^5^ person-years for men and women, respectively [7]. In contrast, in Western countries, ESCC occurs at lower incidence rates (3-20/105 person-years) with a male to female ratio of 8–10:1 [2] and frequently develops in subjects with high combined tobacco and alcohol consumption. ESCC in central Asia often develops in subjects with no smoking and/or drinking history. Risk factors include consumption of hot beverages and deprivation status. Recently, a role for polycyclic aromatic hydrocarbons as potential mutagens in the esophageal mucosa of subjects from Iran has been documented [8]. However, the etiology of ESCC in central Asia is still largely unknown [5].

Independent of geographic origin, molecular changes in ESCC include frequent loss of alleles at chromosomes 3p, 5q, 9p and q, 13q, 17p, 17q or 18q, mutations in tumor suppressor genes such as *TP53*, and genetic and/or epigenetic alterations in *CDKN2a, CCDN1, MYC1 FHIT*, *FEZ1*, *DLC1*, *Annexin-1, CCNB1, TP63, TP73* or *DCC*[9-15]. Increased expression of Epidermal Growth factor Receptor (*Egfr*), sometimes associated with amplification of *EGFR* gene, has been observed in a subset of ESCC [16-18]. *EGFR* and its homolog *HER2* belong to the *ErbB* family of genes encoding transmembrane receptor tyrosine kinase receptors (RTK) which consist of four closely related genes, *EGFR (HER1/ErBb1), HER2 (ErbB2), HER3 (ErbB3)* and *HER4 (ErbB4).* Mutations in the RTK domain of *EGFR* activate the kinase activity by a ligand-independent mechanism. Such mutations are common in adenocarcinomas arising in never-smokers, particularly in women and in patients of Asian origin, and are associated with therapeutic sensitivity to drugs inhibiting the tyrosine kinase (TKIs) [19,20]. However, only few studies have evaluated *EGFR* mutations in esophageal adenocarcinoma [20] or ESCC [17,21-25]. Overall, these reports have identified only rare mutations, with the exception of a recent study focusing on basaloid squamous cell carcinoma subtype in Japanese patients, which reported *EGFR* mutations in 14% of the cases [26].

Here we have analyzed *EGFR* mutations in *EGFR* TK domain (exons 18 to 21) in a total number of 152 ESCC from Iran and India (Kashmir), two areas of the “*Asian Esophageal Cancer Belt”* where smoking and alcohol drinking are not significant risk factors at population level. We hypothesized that, similar to lung cancers of non-smokers, *EGFR* mutations might be more common in this etiological context than in ESCC occurring in the “Western” context of heavy combined tobacco and alcohol use. *HER2* mutations (exons 19 and 20) which have also been observed in a subset of lung cancers of never-smokers [27] and *Egfr* protein expression were also analyzed in a subset of the cases.

## Methods

### Patients

A total of 152 surgically resected or biopsy samples of histologically confirmed ESCC cases were retrieved from pathology archives of hospitals in Iran and India/Kashmir. Cases from Iran (n=98) included 64 biopsies form patients living in Golestan and 34 surgically resected specimens from patients treated in referral centers in Tehran. The cases from Golestan were obtained in the course of the Golestan Case Control study (GCCS, conducted between 2003 and 2007 as a collaborative study between Digestive Disease Research Institute (DDRI), USA National Cancer Institute (NCI) and International Agency for Research on Cancer (IARC, Lyon)) [5,28,29]. They were all analyzed for *EGFR* mutations. The cases from Tehran were from patients treated in three referral centers for esophagectomy (Iran Mehr, Modarres and Madaen Hospitals) between 1991 and 1998. Resected specimens from Tehran were analyzed for *EGFR* mutations and immunohistochemical detection of *Egfr*. The cases from Kashmir Valley included 54 ESCC from patients treated at the Departments of Cardiovascular and Thoracic Surgery and Gastroenterology of the Sher-I-Kashmir Institute of Medical Sciences, Soura, Srinagar, Jammu and Kashmir, between 2002 and 2003. Resected specimens from 17 patients and 37 endoscopic biopsy specimens with confirmed diagnosis of ESCC were included. This series was analyzed for *EGFR* and *HER2* mutations. Informed consent was obtained for GCCS patients. No consent was available for retrospective, archival specimens from Tehran and Kashmir. The study, including anonymized use of archival specimens, was approved by ethical review boards of the DDRI in Iran and the Kashmir Institute of Medical Sciences, Soura, Srinagar Kashmir.

### Mutation analysis

Tumor samples were fixed in 10% buffered formalin, except for a subset of cases from the GCCS which were fixed in 70% ethanol, and all paraffin-embedded. In a previous study, we have shown that there was no bias in DNA extraction and mutation detection between these two fixation methods [30]. Areas with at least 50% tumor cells were selected on 4 μm unstained sections and were scraped off with a disposable scalpel blade. DNA extraction was performed using the QIAamp DNA microkit (QIAGEN, Hilden, Germany). *EGFR* mutation analysis was performed by PCR-based direct sequencing of exons 18 to 21 (tyrosine kinase domain) using primers and annealing conditions as described by Pao *et al.*[31] and Go-Taq polymerase (Promega, Madison, WI, USA). *HER2* was amplified for exons 19 and 20 using the primers and annealing conditions described by Mounawar *et al.*[27]. PCR products were sequenced using Applied Biosystems PRISM dye terminator cycle sequencing method (Perkin-Elmer, Foster City, CA) on ABI PRISM 3100 Genetic Analyzer (Applied Biosystems, Foster City, CA). All PCR products were sequenced in both forward and reverse directions. Presence of mutations was confirmed by a second independent PCR amplification and sequencing.

### Immunohistochemistry

Sections (4 microns) were prepared from paraffin blocks of 34 cases from Tehran, all formalin-fixed. After deparaffinization, inactivation of endogenous peroxidases was done by incubating sections for 40 minutes in 0.3% H_2_O_2_ in methanol. Antigen unmasking was performed in citrate buffer pH 6 for 10 minutes in a high pressure high temperature cooker. Slides were then incubated with mouse monoclonal antibody to *Egfr* (Novocastra, 1/10 dilution) for 1 hour, followed by secondary anti-mouse antibody coupled to peroxidase (Immpress reagent anti-mouse Ig peroxidase, Vector Laboratories, 1/200), diaminobenzidine (DAB)-based revelation and counterstaining with hematoxylin. For each section a negative control was stained without primary antibody. Staining intensity was scored using a four-tier system as defined in Table 1. Samples scored as 2+ or 3+ were considered as “over-expression” [17]. 

**Table 1:** **Definition of Immunostaining scores for *egfr *protein expression in ESCC cases from Tehran (total examined numbers=34)**

**Score**	**Definition**	**Number (%)**
0	No staining or background-type staining	3 (8.8)
1+	Definite cytoplasmic staining and/or equivocal discontinuous membranous staining	9 (26.5)
2+	Unequivocal membrane staining with moderate intensity in over 10%of the cells	16 (47.0)
3+	Strong, continuous membrane staining in over 10% of the cells	6 (17.7)

## Results

A total of 152 cases of primary invasive ESCC samples were analyzed, including 54 specimens from Indian (Kashmiri) patients and 98 specimens from Iranian patients (Table 2). *EGFR* mutations were analyzed by sequencing exons 18 to 21 in all samples. A total of 14 (9.2%) different variations were detected, eight (5.3%) have been reported as known polymorphisms, including a common polymorphism at codon 787 (rs1050171, c.2361 G>A), occurring with a global allelic frequency of 0.417. (http://www.1000genomes.org/ensembl-browser) (Table 3). Four variations (2.6%) were identical to potential or demonstrated activating mutations in *EGFR* TK domain already reported in other cancers, in particular NSCLC (http://www.sanger.ac.uk/genetics/CGP/cosmic/) (Table 4) and two variations (1.3%) have never been reported in any available database (a one-bp deletion in exon 20 (c.2373) and an intronic one (c.2625+68)) (Table 3). These 10 variations were not considered as potential activating *EGFR* mutations.

**Table 2:** Patients’ characteristics

**Characteristics**		**Iran**	**India**	**Total**
		**No (%)**	**No (%)**	**No (%)**
Total No of cases		98	54	152
Age	Mean	63.0	57.0	
	Range	27-84	35-75	
Gender^a^	Male	44 (45.8)	36 (66.7)	80 (46.7)

^a^ Data available for analysis for 150 cases.

**Table 3:** ***EGFR *variations detected in Golestan ESCC cases with unknown impact on TK domain (according to "1000genomes" database)**^**a**^

**Exon/Intron**	**Genomic number**	**Description**	**Frequency of detection**	**SNP ID**
18-Intron	155112	c.2184+100C>T	3	rs17290336
18-Intron	155031	c.2184+19G>A	6	rs17337107
18-Intron	155593	c.2185-98C>T	1^b^	rs62507090
19-Intron	155858	c.2283+69G>A	1^c^	rs17337135
19-Intron	162202	c.2284-60T>C	21	rs10241451
19-Intron	162241	c.2284-21C>T	4	ESP_7_55248965^d^
20-Exon	162339	c.2361A>G	51	rs1050171
20-Exon	162435	c.2457G>A	1	rs56183713
20-Exon	162351	c.2373del1	11	Not reported
21-Intron	172911	c.2625+68C>T	1	Not reported

^a^http://www.1000genomes.org/ensembl-browser.

^b^ This case has also *TP53* mutation at exon 6, codon 211 (G:C>A:T).

^c^ This case has also *TP53* mutation at exon 6, codon 217 (deletion1).

^d^ Reported from NHLBI GO Exome sequencing project.

**Table 4:** **Known activating *EGFR *mutations found in 152 patients (according to COSMIC database)^a^**

**Origin of the patient**	**Tehran**	**India**	**India**	**India**
Mutation type	Missense	Missense	Missense	Microdeletion
Exon No.	19	18	19	19
Nucleotide change	c.2188C>T	c.2156G>A	c.2203C>T	c.2235del15
Amino acid change	p.L730F	p.G719D	p.P753L	p.Q746_A750

^a^http://www.sanger.ac.uk/genetics/CGP/cosmic/.

Among the four activating mutations, three were found in ESCC cases from Kashmir, including one 15 bp deletion in exon 19, (codons 746–750), one missense mutation at codon 719 (exon 18, GGC to GAC, G to D) and one missense mutation at codon 753 (exon 19; CCG to CTG, P to L.). One missense mutation was found in the series from Tehran, located in exon 19 at codon 730 (CTC to CCC, L to P).

Both series have been previously analyzed for *TP53* mutations [30,32,33]. Of the four cases with potential activating *EGFR* mutations, two also carried a mutation in *TP53* (V173G in the case from Tehran; R175H in the case from Kashmir with the G719D *EGFR* mutation).

**Figure 1:**
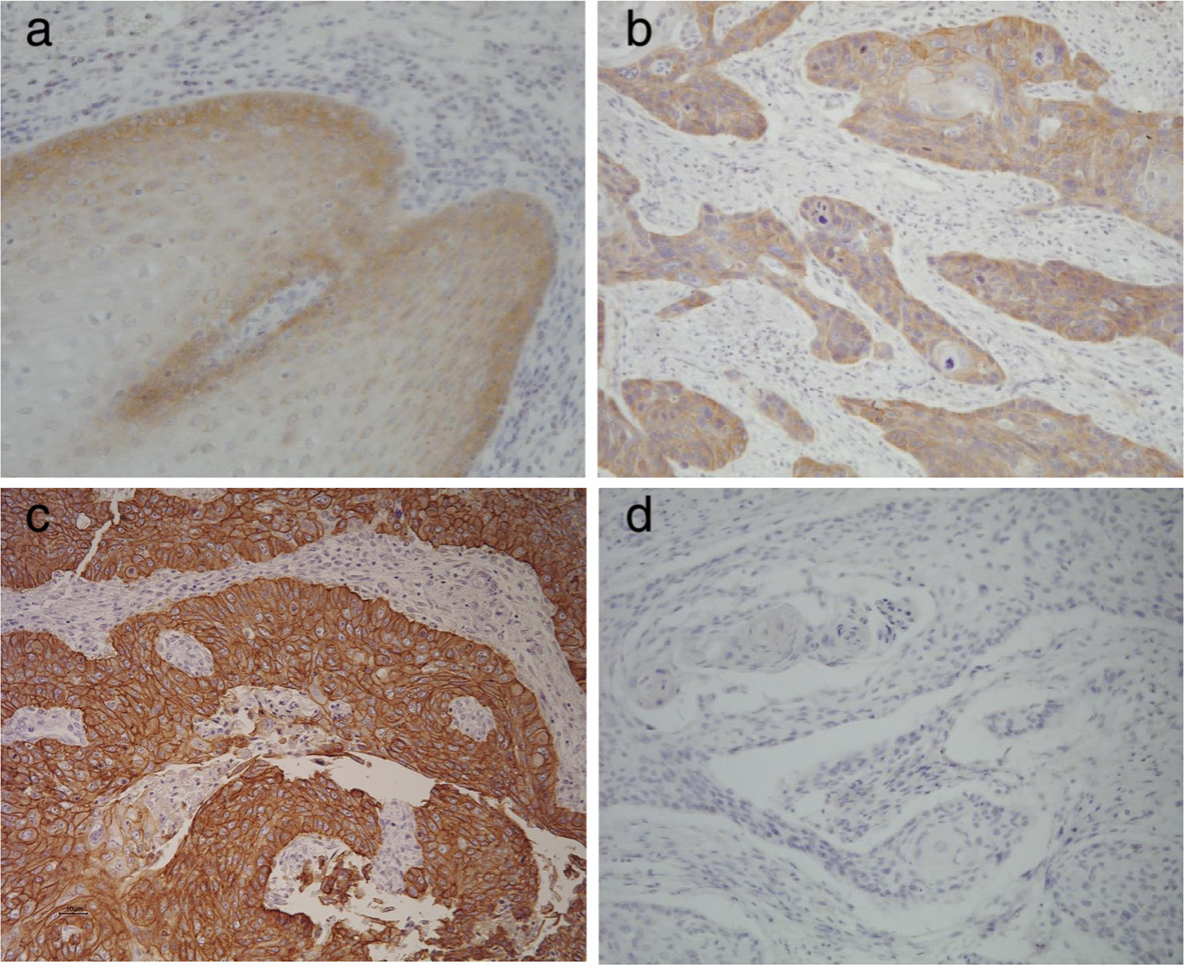
**EGFR expression in normal esophageal epithelium and ESCC.** a-Normal esophageal epithelium with background and weak cytoplasmic staining, scored as 1+; b- ESCC patient from Tehran with *EGFR* mutation (L730F) and protein expression of 2+ positivity; c- ESCC with strong (3+) positive staining in compared with negative normal epithelium; d- negative control. (Magnification ×200).

The series from Kashmir was also analyzed for mutations in exons 19 and 20 of *HER2*, encoding a tyrosine kinase receptor closely related to *EGFR*. No mutation was found. *Egfr* expression status was analyzed by immunohistochemistry in the series from Tehran. Over-expression (staining scores 2+ or 3+) was detected in 65 % of the cases (22 of 34) whereas 26 % (9 of 34) were scored as 0 and 9% (3 of 34) were scored as 1+, comparable to staining intensity in normal epithelium (Table 1). Among mutated cases, only the one with L730P *EGFR* mutation showed *Egfr* over-expression and was scored as 2+ (Figure 1).

## Discussion

Mutations in *EGFR* have attracted attention because of their common occurrence in lung cancers of non-smokers (in particular in adenocarcinoma, women, and patients of Asian origin) and because of their significance as predictors of response to therapeutic tyrosine kinase inhibitors [19,34,35]. In lung cancer, *EGFR* mutations cluster in exons 18 to 21, encoding the domain of the tyrosine kinase that contains the ATP binding pocket. The most common mutations are short, in frame deletions in exon 19 (34%) and missense mutations at codon 858 (p.L858R) in exon 21 (36%) (http://www.sanger.ac.uk/genetics/CGP/cosmic/). These mutations modify the geometry of the ATP binding cleft in the tyrosine kinase, resulting in a hyperactive form of the receptor. These are not restricted to lung adenocarcinoma and we have reported two mutations among four lifetime never-smokers with squamous cell carcinoma of the lung [27]. Mutations are infrequent in other cancer pathologies analyzed to date [20,36].

Based on the hypothesis that these mutations may preferentially occur in a context of non-tobacco dependent carcinogenesis, we investigated whether *EGFR* mutations could be detected in three series of ESCC from central Asia (two high incidence areas in Northern Iran (Golestan) and Northern India (Kashmir) and one low incidence area in Iran (Tehran). The etiology of ESCC in these areas has been addressed in a number of studies [5,7,8,28,37,38]. Overall, epidemiological studies have consistently reported that tobacco usage is not a significant risk factor, in contrast with ESCC detected in most Western countries and in Japan. Sequencing of exons 18 to 21 of *EGFR* in a total of 152 ESCC cases detected 14 variations (9.2%). Comparison with COSMIC database indicates that four of these variations are known activating mutations in *EGFR* TK domain (2.6%), including a common deletion (p.Q746_A750del; 560 occurrences in the COSMIC database) and three less common missense mutations. The other variations identified in this study are known polymorphisms except for two unknown never reported variations which is not clear whether they may activate the tyrosine kinase in the same way as well-characterized *EGFR* activating mutations.

Previous studies have shown that *EGFR* activating mutations were rare in ESCC. In a study of 57 cases, Guo *et al.* reported three *EGFR* mutations, including a truncating mutation at E872 and two silent mutations at G873 and P753 [23]. In another series of 40 cases (15 of which with amplification of *Egfr*), Hanawa *et al.* did not detect any mutation in exon 19 or 21 [17]. To our knowledge, the only study in which a significant proportion of cases contained *EGFR* mutation (14%) was focused on a rare variant form, basaloid squamous cell carcinoma, suggesting that aberrations in *EGFR* may be involved in this particularly aggressive form of the disease [26]. Notably, the series analyzed here does not include basaloid forms of ESCC but comprises cases from Golestan, an area in which we have previously reported an extremely high rate of *TP53* mutations (90%), suggestive of the role of diverse mutagens in esophageal carcinogenesis [30]. Thus, contrary to our hypothesis, and regardless of involvement of environmental mutagens in ESCC from Golestan, *EGFR* mutations in non-smoking ESCC patients appears to be a rare event which may not play a significant role in the pathogenesis of ESCC. Further studies are needed to determine such *EGFR* mutations tend to occur at higher frequency in specific groups of ESCC patients.

Despite infrequent mutations in *EGFR*, over expression of the protein, with or without gene amplification, is a relatively frequent event in ESCC [16-18,39-41]. In 2006, Hanawa *et al.* reported that, among 53 cases of ESCC with high protein expression levels, FISH analysis of amplification revealed clear amplification in 15 cases, evidence for modest changes in copy numbers in 32 cases and no evidence for amplification in six cases, clearly showing that amplification is only partially correlated with expression [17]. In the present series, we detected *Egfr* over expression in 65% of the cases, irrespective of tumor grade and stage. However, we have not analyzed the amplification status of the *EGFR* locus. The only case with known *EGFR* activating mutation expressed *Egfr* at moderate levels. The frequent over-expression of *Egfr* has led to the concept that *Egfr*-targeting therapies may have some benefit in patients with advanced esophageal cancer. A phase II study of Gefinitib, an *Egfr* tyrosine kinase inhibitor, in second-line treatment of advanced esophageal cancer, reported that a significantly higher disease control rate (response plus stable disease) was observed in patients with ESCC histology or with high *Egfr* expression [42]. These results concur with ours to suggest that further efforts should be made to select esophageal cancer patients who may benefit from *Egfr*-targeted therapies.

## Conclusions

This report is the first report for identification of *EGFR* activating mutations in ESCC. Previous studies failed to identify such mutations. Focusing our analysis on groups of non-smoking patients from Asia, we found rare activating mutations (4/152 cases, 2.6%) but frequent *Egfr* protein over-expression (65%). These results suggest that further efforts should be developed to determine whether *EGFR* mutations occur in specific groups of ESCC patients and whether these esophageal cancer patients may benefit from *Egfr-*targeted therapies.

## Abbreviations

EGFR: Epidermal growth factor receptor; SCC: Squamous cell carcinom; ESCC: Esophageal squamous cell carcinoma; TK: Tyrosine kinase; TKR: Tyrosine kinase receptor; EGF: Epidermal growth factor; NSCLC: Non-small cell lung cancer; IHC: Immunohistochemistry; GCCS: Golestan case–control stud; DDRC: Digestive disease research center; NCI: National cancer institute; IARCm: International agency for research on cancer; GEMINI: Gastroesophageal malignancies in northern Iran.

## Competing interests

The authors declare that they have no competing interests.

## Authors’ contributions

BAA carried out most of the laboratory experiments and all the pathology evaluation, prepared the manuscript draft. NAD carried out some part of the laboratory experiment, helped to draft the manuscript. MMM provided part of the samples and critically reviewed the manuscript draft. SAZ provided some part of samples and critically reviewed the manuscript draft. MML provided some part of samples and critically reviewed the manuscript draft. GMP supervised the laboratory experiments. SV analyzed the sequencing data. RM participated in the study design. MM supervised some part of the laboratory experiments. FS participated in the study design. PH designed, coordinated and supervised the study and critically reviewed the manuscript draft. All authors read and approved the final manuscript.

## Pre-publication history

The pre-publication history for this paper can be accessed here:

http://www.biomedcentral.com/1471-2407/12/602/prepub
